# Internet addiction and its relationship with attention deficit hyperactivity disorder (ADHD) symptoms, anxiety and stress among university students in Malaysia

**DOI:** 10.1371/journal.pone.0283862

**Published:** 2023-07-28

**Authors:** Hazli Zakaria, Imran Hussain, Nor Sa’adah Zulkifli, Norazimah Ibrahim, Nuri Jailina Noriza, Michelle Wong, Nik Ruzyanei Nik Jaafar, Hajar Mohd. Salleh Sahimi, Muhammad Hanif Abd Latif

**Affiliations:** 1 Department of Psychiatry, Faculty of Medicine, Hospital Canselor Tuanku Muhriz, Universiti Kebangsaan Malaysia, Cheras, Kuala Lumpur, Malaysia; 2 Department of Otolaryngology, Hospital Queen Elizabeth, Sabah, Malaysia; 3 Department of Anaesthesiology, Hospital Raja Permaisuri Bainun (HRPB) Ipoh, Perak, Malaysia; 4 Department of Anaesthesiology, Hospital Jasin, Melaka, Malaysia; 5 Klinik Kesihatan Beladin, Pusa, Sarawak, Malaysia; 6 Klinik Kesihatan Bawang Assan, Sibu, Sarawak, Malaysia; University of Catania Libraries and Documentation Centre: Universita degli Studi di Catania, ITALY

## Abstract

**Background and aims:**

There is growing evidence on the contribution of psychological factors to internet addiction; yet it remains inconsistent and deserves further exploration. The aim of this study was to determine the relationship between the psychological symptoms (Attention Deficit Hyperactivity Disorder (ADHD) symptoms, stress, depression, anxiety and loneliness) and internet addiction (IA) among the university students in Malaysia.

**Materials and methods:**

A total of 480 students from different faculties in a Malaysian public university participated in this study. They were selected by simple random sampling method. They completed self-administered questionnaires including the Malay Version of Internet Addiction Test (MVIAT)) to measure internet addiction and Adult Self-Report Scale (ASRS) Symptom Checklist, Depression Anxiety Stress Scales (DASS) and UCLA Loneliness Scale (Version 3) to assess for ADHD symptoms, depression, anxiety, stress, and loneliness respectively.

**Results:**

The prevalence of IA among university students was 33.33% (n = 160). The respondents’ mean age was 21.01 ± 1.29 years old and they were predominantly females (73.1%) and Malays (59.4%). Binary logistic regression showed that gender (p = 0.002; OR = 0.463, CI = 0.284–0.754), ADHD inattention (p = 0.003; OR = 2.063, CI = 1.273–3.345), ADHD hyperactivity (p<0.0001; OR = 2.427, CI = 1.495–3.939), stress (p = 0.048; OR = 1.795, CI = 1.004–3.210) and loneliness (p = 0.022; OR = 1.741, CI = 1.084–2.794) were significantly associated with IA.

**Conclusion:**

A third of university students had IA. In addition, we found that those who were at risk of IA were males, with ADHD symptoms of inattention and hyperactivity, who reported stress and loneliness. Preventive strategy to curb internet addiction and its negative sequelae may consider these factors in its development and implementation.

## Introduction

The internet has become part of our lives nowadays, as it connects the world and eases the lives of humankind. Nevertheless, the increasing use of the internet has led to increasing incidence of internet addiction (IA) especially among the youth. IA is defined as any online-related, compulsive behaviour which interferes with normal living and causes severe stress on family, friends, loved ones, and one’s work environment [1]. Particularly in the last two decades, IA has risen to become a global concern to the public [1]. A local study amongst selected secondary school students in Malaysia found that 49.2% of students were addicted to the internet [2]. During the recent COVID pandemic, a high prevalence rate of IA (83.5%) was identified among medical students in a Malaysian public university where its high usage may serve as a coping method against anxiety during the pandemic [3]. The department of statistics Malaysia reported that 91.7% Malaysian households will be using the internet in 2020 [4], with a 155% increase in children aged 5–17 years old using the internet from 2016 to 2020 [5]. The prevalence of problematic Internet users in Malaysia could be as high as 37% to 43.9% [6, 7]. 23% among school aged children were addicted to the internet, with users spending 2 hours per day during school holiday [8].

Many psychological factors have been associated with IA. Secondary school students with severe anxiety and depression scores were found to be addicted to the internet [2]. IA was also identified as a significant predictor for depression, anxiety, stress and suicidality [9]. A systematic review by Wang et al. (2017) found that attention deficit/hyperactivity disorder (ADHD) was positively correlated with IA among adolescents and young adults [10]. People with ADHD are vulnerable to IA due to impaired inhibition leading to lack of self-control and difficulties in self-restraint [10, 11]. Those with psychological symptoms such as feeling lonely, depressed or anxious may use the internet to cope as it offers many activities that could be accessed simultaneously, and one would gain immediate reward which may alleviate boredom and perhaps loneliness [12].

IA may potentially pose a threat to one’s health and social well-being. It can lead to procrastination, lower productivity, reduction in social life and physical symptoms such as backache and neck pain [13, 14], and fatigue [15] and the ill effect of sedentary lifestyles including overweight and obesity [16, 17]. A study by Ercan et al. (2021) explained that IA influences body mass index leading to obesity, poor sleep quality and attention and memory problems in daily life [18]. Poor academic performances among medical students were reported to be associated with IA and this finding was supported by another study conducted locally among Malaysian university students [19, 20]. Leung & Lee (2012) found that adolescents with IA often missed their classes and had low social skills which affected their academic performance [21]. In addition, IA had a negative impact on effort, dedication and energy to study among university students [22]. A local study by Ambad et al. (2017) that was conducted to determine the impact of IA on academic performance had concluded a bidirectional relationship between IA and emotional instability. Negative feelings such as stress resulted in emotional instability that led to excessive usage of the internet which had a significant impact on a student’s academic performance. Furthermore, IA itself causes emotional instability that could lead to depression [23]. Severity of internet use disorder was positively linked to depression, hoarding and obsessing symptoms [24].

Although the effects of IA have been extensively studied, the psychosocial profile contributing to IA is still under explored in Malaysia. This study aimed to determine the prevalence of IA and to determine the associations of socio-demographic and psychological factors (specifically ADHD symptoms, stress, depression, anxiety, and loneliness) with IA among university students.

## Methodology

### Study design and data collection

This study recruited students from the faculties of dentistry, medicine, optometry, pharmacy, and psychology of University Kebangsaan Malaysia (UKM), a public university in Malaysia. Data were collected cross-sectionally between February to May 2016. Based on the similar study, the sample size was calculated using the formula of prevalence, n = (Z1-α)2(P(1-P)/d2), with level of accuracy (d) taken as 0.05, and confidence level of 95%, at least 384 samples required for this study [25].

The inclusion criteria included: (1) UKM students, (2) aged between 18 and 24 years old, (3) Malaysian’s citizen, and (4) consented to participate in the research. There were no exclusion criteria for this study. Participants were approached after their classes with the permission of their lecturers. Those who voluntarily agreed to participate were explained the study’s procedures, purposes, participation benefits and risks, and assurance of anonymity, as well as their right to withdraw from the study at any point of time; before signing a written informed consent form. Those who provided consent were asked to complete the questionnaires. This study was approved by the Medical Research Committee of Universiti Kebangsaan Malaysia Medical Centre and it abides by the regulations of the 1964 Declaration of Helsinki and its subsequent amendments.

### The instruments

#### Demographic data

This questionnaire contains demographic variables of the subjects which included the participant’s age, gender, race and religion.

#### Malay Version of Internet Addiction Test (MVIAT)

The Malay Version of Internet Addiction Test (MVIAT) is a reliable and valid measure of addictive use of Internet, originally developed by Dr Kimberly Young [7, 26]. It consists of 20 items that measures mild, moderate and severe level of Internet Addiction. The questions need to be answered by using the response scale from 1 to 5 (1 = never to 5 = always) reflecting the frequency of the symptoms. The questionnaire also evaluate the frequency of functional impairment such as deterioration in performing household chores, school work and job performance due to internet use. The higher the scores, the greater the level of addiction and problems resultant from the internet usage. The cut-offs used are those with scores between 0 and 48 is considered as having no internet addiction while 49 and more is considered as having internet addiction. The instrument showed good internal consistency with Cronbach’s α = 0.91 [7].

#### Adult Self-Report Scale (ASRS) symptoms checklist

The Adult Self-Report Scale (ASRS) Symptom Checklist is a measurement tools to assess the ADHD symptoms which basically has two components that is inattentive (Part A) and hyperactive or impulsive (Part B) [27]. If the score is at least 17 for either Part A or Part B, the patient has symptoms consistent with ADHD and a more thorough clinical evaluation to understand impairments and history is warranted. ASRS has acceptable internal consistency ranging from 0.63 to 0.72 [28].

#### Depression Anxiety Stress Skills (DASS)

The DASS is a set of three self-report scales designed to measure the negative emotional states of depression, anxiety and stress [29]. It is a 42-item questionnaire. Each of the three DASS scales contains 14 items, divided into subscales of 2–5 items with similar content. The Depression scale assesses dysphoria, hopelessness, devaluation of life, self-deprecation, lack of interest/involvement, anhedonia, and inertia. The Anxiety scale assesses autonomic arousal, skeletal muscle effects, situational anxiety, and subjective experience of anxious affect. The Stress scale is sensitive to levels of chronic non-specific arousal. It assesses difficulty relaxing, nervous arousal, and being easily upset/agitated, irritable/over-reactive and impatient. Subjects are asked to use 4-point severity/frequency scales to rate the extent to which they have experienced each state over the past week. Scores for Depression, Anxiety and Stress are calculated by summing the scores for the relevant items. The cut-offs used are more than 9(depression), more than 7 (anxiety) and more than 14 (stress) [30]. It has revealed excellent Cronbach’s alpha values of 0.94, 0.90 and 0.87 for depressive, anxiety and stress domains respectively [31].

#### UCLA loneliness scale (Version 3)

This is a 20-item scale designed to measure one’s subjective feelings of loneliness and social isolation. Participants rate each item on a scale from 1 (Never) to 4 (Always). The scoring is on a continuous basis with the higher scores indicate greater degrees of loneliness. This scale was reported to have good reliability and validity [32]. The cut-off used is more than 49 (loneliness). It also has good internal consistency with Cronbach’s alpha ranging from 0.89 to 0.94 [32].

### Statistical analysis

The results were analysed using the Statistical Package for Social Sciences, version 27.0. Odd ratio and Chi-square test were used to evaluate the association between independent variables with internet addiction; and binary logistic regression analysis was used to evaluate the most significant factor that contribute to internet addiction after controlling for confounding factors.

## Results

A total of 480 of university students participated in this study and about a third of them (n = 160; 33.3%) were considered to have internet addiction. The sociodemographic and psychological variables were summarized in Table 1. The participants’ age ranged between 18 to 24 years old with the mean age was 21.01 ± 1.29 years old. They were predominantly females (73.1%) and Malays (59.4%). The other ethnicities of the sample participants were as follows: Chinese (25.8%), Indian (11.3%), and other ethnic groups (3.5%); which reflected the ethnic distribution of Malaysia.

**Table 1:** Description of the participants’ sociodemographic and psychological variables.

Variables	Categories	Frequency (n = 480)	Percentage (%)
**Age (mean±S.D.)**	18–24 (21.01± 1.29 years old.)		
**Gender**	Male	129	26.9
	Female	351	73.1
**Race**	Malay	285	59.4
	Chinese	124	25.8
	Indian	54	11.3
	Others	17	3.5
**Religion**	Islam	302	62.9
	Buddha	104	21.7
	Hindu	43	9.0
	Christian	27	5.6
	Others	4	0.8
**ADHD (Inattention)**	Likely	233	48.5
	Unlikely	247	51.5
**ADHD (Hyperactivity)**	Likely	159	33.1
	Unlikely	321	66.9
**Depression**	Depressed	157	32.7
	Normal	323	67.3
**Anxiety**	Anxious	249	51.9
	Normal	231	48.1
**Stress**	Stressed	149	31.0
	Normal	331	69.0
**Loneliness**	Lonely	161	33.5
	Normal	319	66.5

For the association between IA and the variables studied, females were found to be significantly associated with IA p<0.001) together with all the domains of the psychological symptoms. The presence of ADHD symptoms was divided into two domains: inattention and hyperactivity. Each of the symptom domains was then classified to be unlikely and likely to have ADHD. Of those with IA, 71.2% and 54.4% of them were likely to have ADHD inattention and hyperactivity respectively, which were statistically significant (p<0.001 for both domains). Other psychological factors that were significantly associated with IA were: depression (p<0.001), anxiety (p<0.001), stress (p<0.001) and loneliness (p<0.001), as shown in Table 2.

**Table 2:** The association between internet addiction and psychosocial variables.

Risks	Categories	Internet Addiction		*p* value	OR	95% Confidence interval	
		Addicted	Normal			Lower	Upper
**Gender**	Male	60(37.5%)	69(53.5%)	**<0.001**	2.183	1.439	3.310
	Female	100(62.5%)	251(71.5%)				
**Race**	Malay	99(61.9%)	186(65.3%)	0.430	1.169	0.793	1.725
	Non-malay	43(38.1%)	134(68.7%)				
**Religion**	Muslim	109(68.1%)	193(63.9%)	0.095	1.406	0.942	2.100
	Non- Muslim	51(31.9%)	127(71.3%)				
**ADHD** Inattention	Likely	114 (71.2%)	119 (51.1%)	**<0.001**	4.186	2.776	6.311
**ADHD** Hyperactivity	Likely	87 (54.4%	72 (45.3%)	**<0.001**	4.105	2.733	6.167
**Depression**	Depressed	88 (55.0%)	69 (43.9%)	**<0.001**	4.446	2.951	6.698
**Anxiety**	Anxious	115 (71.9%)	134 (53.8%)	**<0.001**	3.547	2.354	5.345
**Stress**	Stressed	85 (53.1%)	64 (43%)	**<0.001**	4.533	2.996	6.859
**Loneliness**	Lonely	78 (48.7%)	83 (51.6%)	**<0.001**	2.716	1.824	4.045

Table 3 referred to the subsequent binary logistic regression analysis which showed that depression (p = 0.189) and anxiety (p = 0.212) became insignificant when confounders were controlled in this study. Only gender (p = 0.002), both ADHD components of inattention (p = 0.003) and hyperactivity (p<0.001), loneliness (p = 0.022) and stress (p = 0.048) were found to have significant relationship with IA. ADHD symptoms of hyperactivity and inattention were found to be the biggest predictors of IA as those with hyperactivity had 2.4 times greater likelihood to have IA (p<0.001; OR = 2.427, CI = 1.495–3.939), while those ADHD symptoms of inattention were twice more likely to have IA (p = 0.003; OR = 2.063, CI = 1.273–3.345). Other significant predictors were stress (p = 0.048; OR = 1.775, CI = 1.004–3.210) and loneliness (p = 0.022; OR = 1.741, CI = 1.084–2.794).

**Table 3:** Binary Logistic Regression showed the relationship between internet addiction and the psychosocial variables.

		β	SE	*P* value	OR	95% Confidence interval	
						Lower	Upper
Risks	Gender	-0.771	0.249	**0.002**	0.463	0.284	0.754
	ADHD Inattention	0.724	0.246	**0.003**	2.063	1.273	3.345
	ADHD Hyperactivity	0.887	0.247	**0.000**	2.427	1.495	3.939
	Depression	0.393	0.299	0.189	1.481	0.824	2.661
	Anxiety	0.351	0.281	0.212	1.421	0.819	2.466
	Stress	0.585	3.896	**0.048**	1.795	1.004	3.210
	Loneliness	0.554	5.269	**0.022**	1.741	1.084	2.794

## Discussion

This study found a high prevalence of IA among university students which was 33.3%. This result lies within the range and comparable to the studies conducted using the same scale which found a prevalence of 37% [6, 7, 33]. The Malay version of IAT scale is, to the authors’ knowledge, the only validated tool available in Malay to screen for internet addiction up to this point [7]. The former study measured IA among the adolescents while this study assessed IA in young adults who were attending university. Indeed, educational attainment was deemed a vital indicator of internet access in Malaysia [5]. A large proportion of the internet users (55.6%) who were still studying; were those who attended college or university [5]. Nevertheless, high proportion of the young group who used the internet excessively is rather disturbing as the internet could lead to physical symptoms, mental health problems including anxiety, depression and other psychopathological symptoms [34, 35]; together with social consequences such as lower recreational activities, poor academic performance and social withdrawal [36, 37].

This study also found a gender predisposition towards IA which concurs with the previous study findings i.e. there was an overall greater risk of IA among males [38]. The mechanisms involved are multifactorial and may be complex including genetic [39], hormonal factors, emotion or other psychological factors [40], perception of self-image, environmental and sociocultural factors [41]. Interestingly, it was observed that the discrepancy may not be as wide as before in several of the recent studies; some studies even found a female predominance in internet use [42, 43]. Understandably, over the decades, the internet has evolved as a tool of communication and information. The internet may serve not only the basic but more complex human needs in connecting people and other activities including entertainment, shopping and work that influenced the behaviors of the internet utilisation by the different genders [44].

Among the psychological factors that were examined in this study, ADHD symptoms, both inattention and hyperactivity; were the most significantly linked to IA with a greater than two-fold risk. A study by Concerto et al. (2021) found that ADHD symptoms were positively associated with severity of internet gaming disorder in adults [45]. The nature of the internet is multi-modal, fast paced with almost instantaneous response, and the games’ high stimulation may match the vulnerability of youth with ADHD [46]. Lack of external stimulation among ADHD individuals was significantly correlated with higher internet gaming engagement [47]. Most games incorporate multiple reinforcement strategies that continue to attract individual players to engage more and more to the game. This provides strong incentives to individuals with ADHD [48]. Moreover, the swiftly changing screens put marginal demands on attention and working memory [49], enforced effort, or writing [50] all of which are challenging in ADHD. A meta-analysis concurred the significant association between IA and ADHD and a variety of psychiatric morbidities including alcohol abuse, depression and anxiety [51]. Stress is another psychological factor that is found to influence IA. This study showed that those who reported stress had almost twice the risk of having IA. Some studies had linked IA with stress, depression and anxiety [52] and a few studies found only depression and stress to be significantly correlated with internet addiction [53, 54].

A more recent systematic review had found high prevalence of depression in people with internet gaming disorder with approximately one out of three participants affected with more severe depressive symptoms compared to the general population [55]. In our study, only stress was found significantly associated with IA. Depression, anxiety and stress have been proposed to affect a person’s activities, work, and studies, as well as influencing decision-making and coping mechanisms [56–58]. This study was conducted on university students who understandably would experience a fair level of stress in their academic pursuit. Engaging with the internet is an avoidance in coping with the stress as the internet distracts an individual from their actual tasks [59]. Identified psychosocial factors to moderate depression includes 1) avoidant coping style [60]; possibly through excessive use of the internet; and 2) campus connectedness; which is particularly relevant among the university students [60]. These variables possibly buffer the relationship between stress and clinical disorders like depression and anxiety.

Similar to stress, those who reported loneliness had almost twice the risk of IA. The internet serves as an avenue for escapism from real problems like loneliness, helplessness, and boredom [12]. Therefore, those who reported loneliness are more likely to be at risk of IA. Loneliness may give rise to IA from the human’s need to socially interact with another human being; whereby individuals may choose to express their emotions and thoughts to others through the internet and avoid face-to-face communication [61]. However, the relationship can be bi-directional. One may also argue that the excessive use of the internet leads to reduced social connections outside the net and social withdrawal [62, 63]; both of which lead to loneliness.

## Limitation

The main limitations in this study include 1) it has limited generalizability to the Malaysian population as this study drew inference from one university in an urban setting, 2) it is a cross-sectional study; which could not imply causality in the relationship between IA and the associated psychological factors, and 3) participants who already had the diagnosis of ADHD/depression or anxiety were not excluded which could distort the results.

## Conclusion

This study concludes that one in three of the university students was addicted to the internet. It identified the factors that posed greater likelihood to have internet addiction which include males, having ADHD symptoms (either inattention or hyperactivity; or both), stress and loneliness. The interactions between IA and psychological factors are complex and could potentially lead to substantial health burden in relation to young adults. It is recommended that future studies to examine the cause-and-effect and explore their in-depth pathways, in addition to determine the best preventive strategies particularly on the vulnerable group to curb the negative sequelae of internet addiction.
